# Identifying treatment effects of an informal caregiver education intervention to increase days in the community and decrease caregiver distress: a machine-learning secondary analysis of subgroup effects in the HI-FIVES randomized clinical trial

**DOI:** 10.1186/s13063-020-4113-x

**Published:** 2020-02-14

**Authors:** Megan Shepherd-Banigan, Valerie A. Smith, Jennifer H. Lindquist, Michael Paul Cary, Katherine E. M. Miller, Jennifer G. Chapman, Courtney H. Van Houtven

**Affiliations:** 1 Durham VA HSR&D ADAPT, Durham VA Health Care System, 508 Fulton Street, Durham, NC 27705 USA; 2 Department of Population Health Sciences, Duke School of Medicine, 215 Morris Street, Durham, NC 27701 USA; 3 Division of General Internal Medicine, Department of Medicine, Duke University School of Medicine, 300 Morris Street, Durham, NC 27701 USA; 4 School of Nursing, Duke University, 307 Trent Drive, Durham, NC 27710 USA; 5 Department of Health Policy and Management, University of North Carolina-Chapel Hill, 135 Dauer Drive, Chapel Hill, NC 27599-7400 USA

**Keywords:** Family caregiver intervention, Institutionalization, Caregiver depression, Clinical trial, Heterogeneous treatment effects

## Abstract

**Background:**

Informal caregivers report substantial burden and depressive symptoms which predict higher rates of patient institutionalization. While caregiver education interventions may reduce caregiver distress and decrease the use of long-term institutional care, evidence is mixed. Inconsistent findings across studies may be the result of reporting average treatment effects which do not account for how effects differ by participant characteristics. We apply a machine-learning approach to randomized clinical trial (RCT) data of the *Helping Invested Family Members Improve Veteran’s Experiences Study* (HI-FIVES) intervention to explore how intervention effects vary by caregiver and patient characteristics.

**Methods:**

We used model-based recursive partitioning models. Caregivers of community-residing older adult US veterans with functional or cognitive impairment at a single VA Medical Center site were randomized to receive HI-FIVES (*n* = 118) vs. usual care (*n* = 123). The outcomes included cumulative days *not* in the community and caregiver depressive symptoms assessed at 12 months post intervention. Potential moderating characteristics were: veteran age, caregiver age, caregiver ethnicity and race, relationship satisfaction, caregiver burden, perceived financial strain, caregiver depressive symptoms, and patient risk score.

**Results:**

The effect of HI-FIVES on days not at home was moderated by caregiver burden (*p* < 0.001); treatment effects were higher for caregivers with a Zarit Burden Scale score ≤ 28. Caregivers with lower baseline Center for Epidemiologic Studies Depression Scale (CESD-10) scores (≤ 8) had slightly lower CESD-10 scores at follow-up (*p* < 0.001).

**Conclusions:**

Family caregiver education interventions may be less beneficial for highly burdened and distressed caregivers; these caregivers may require a more tailored approach that involves assessing caregiver needs and developing personalized approaches.

**Trial registration:**

ClinicalTrials.gov, ID:NCT01777490. Registered on 28 January 2013.

## Background

Maintaining aging adults at home is an important policy goal [1]. Informal caregiving, or providing unpaid care for a family member or friend, can substitute costly institutional-based long-term care [2, 3]. However, informal caregivers often report high levels of burden and depressive symptoms [4] which may lead to patient placement in institutional care [5]. Strengthening caregiver skills, support, and connection to health system resources, can reduce burden [6, 7] and psychological symptoms [8] and improve the ability of caregivers to care for patients at home [9–11]. However, systematic reviews of interventions for caregivers of multiple patient populations show mixed results [12–16]. By and large these systematic reviews were rigorously designed and included randomized controlled trial (RCT) design studies which lend credence to these results. Therefore, it is possible that inconsistencies in outcomes across studies are related to the composition of the study samples. For example, within a study sample the treatment effect may be different for specific subgroups than for the overall sample [17]. A recent randomized clinical trial of a nine-session education intervention, *Helping Invested Family Members Improve Veteran’s Experiences Study* (HI-FIVES), for caregivers of veterans who were functionally impaired did not identify an average treatment effect on days in the community or caregiver depressive symptoms. The median of days not at home for participants randomized to HI-FIVES was 3 days vs. 3 days for control (i.e., usual care) participants while the mean of days not at home for HI-FIVES participants was 8.9 (SD = 13) vs. 6 (SD = 14.5) for control. At 12 months post baseline, caregivers in HI-FIVES had a mean Center for Epidemiologic Studies Depression Scale (CESD-10) score of 8.2 (SD = 6.6) vs. 7.6 (SD = 5.6) for the usual care group [18]. However, a subsequent study using HI-FIVES data examined the data for hypothesis-driven subgroup effects. This study found that hospitalization risk moderated the effect of HI-FIVES; Veterans with a medium vs. high hospitalization risk spent more days at home as a result of the HI-FIVES intervention [19].

However, it is possible that there remain systematically different outcomes among subgroups not identified a priori. Traditional statistical tests to identify effects among multiple subgroups are underpowered and susceptible to multiple testing errors because they consider one factor at a time [20]. Further, it is possible that combinations of characteristics, such as race/ethnicity and income, rather than single characteristics, give rise to these heterogeneous treatment effects—or differences in treatment effect by subgroup [21]. To address these limitations, we apply machine-learning methods for identifying heterogeneous subgroups to understand which less discernible subgroups might benefit (or not) from the HI-FIVES intervention. Unlike traditional regression models which employ pre-specified structural hypotheses, machine learning seeks patterns in the data to identify important predictors and predictor interactions and are thus a preferable approach when the research questions seek to discover associations rather than test a priori hypotheses. Specifically, we apply model-based recursive partitioning methods to data from HI-FIVES [22] to examine the effect of predictors of treatment effects across subgroups simultaneously, which is likely a more accurate portrayal of how individual-level characteristics operate together to compound the benefits or risks of treatment. In addition, this approach avoids multiple testing errors by building a decision tree through iteratively partitioning a space that comprises multiple covariates. In contrast, standard variable by variable interaction methods are only able to partition the space of one covariate at a time which increases multiple testing error when analysts need to examine multiple potential moderators [23].

The objective of this study is to examine whether the average treatment effect of the HI-FIVES trial masked treatment effects among subgroups of the trial sample. This additional step of post-trial subgroup testing is important for future interventions designed to target the needs of dyads who might receive beneficial effects [6, 14].

## Methods

This study adheres to Consolidated Standards of Reporting Trials (CONSORT) guidelines.

### Aim and study design

We applied machine-learning methods to conduct a post-hoc analysis of the HI-FIVES trial data to explore whether treatment effects varied within subgroups of caregivers. Specifically, we tested for heterogeneous treatment effects of HI-FIVES, a RCT of a caregiver education intervention [18] on days not at home and caregiver depressive symptoms among a sample of informal caregivers.

### Participants

Informal caregivers of patients who received a referral for Veteran Health Administration (VHA) home and community-based services (HCBS) or geriatric clinics in the prior 6 months were identified through telephone contact with the patient (*n* = 3746). Note that individuals referred for obesity, diabetes, blood pressure care or temporary care only were removed from the potential sample. Both the patient and caregiver had to qualify for the study. Ineligibility criteria for patients included (1) referral to nursing home care or hospice in the past 6 months, (2) currently residing in an institution or hospital, (3) identified as being fully independent, (4) unable to communicate in English, (5) having no telephone number, (6) no identified informal caregiver, and (7) the presence of a behavioral flag in the medical records. Caregivers were ineligible if they were: (1) under age 18 years, (2) could not commit to attending four weekly group sessions, (3) currently participating in another caregiver study, and (4) having five or more errors on the Short Portable Mental Status Questionnaire (SPMSQ). A total of 241 total dyads were consented and enrolled by the study research assistant [18]. See Van Houtven, et al. for the CONSORT Diagram [18]. Dyads were stratified by patient cognitive status and whether the patient was a high health care utilizer and within the strata participants were randomly allocated 1:1 to two arms (HI-FIVES intervention vs. usual care) via a computer-generated randomization sequence. The study biostatistician conducted the randomization procedure. High health care utilizer was defined as an individual with two or more unique inpatient hospitalizations in the year prior to the most recent date of referral. Dyads in the treatment arm (*n* = 118) received a nine-session caregiver education intervention while dyads randomized to usual care (*n* = 123) received routine services offered through the HCBS referral process. All caregivers received information about the Veterans Affairs (VA) Caregiver Support Program (Public Law 111–163).

### Intervention

HI-FIVES comprised three weekly individual telephone training calls to the caregiver to improve behaviors related to medication management and four additional topics chosen by the caregiver [18, 24]. Topics included content such as rewards and frustrations of caregiving, clinical care, self-care, navigating the VA, planning for the future, and resources for caregivers. Following the telephone training calls, caregivers participated in four weekly group education sessions lead by the interventionist and a VA caregiver support coordinator to address common issues facing caregivers of complex patients. Caregivers also received two individual-level booster calls 1 and 2 months after completion of the group sessions.

### Outcome measures

Our study considered two outcomes. The first outcome was the number of days the veteran was *not* at home (e.g., in emergency department (ED), hospital or post-acute facility) during the 12 months following randomization; institutional hospice stays were not included as days not at home. This outcome was assessed using VA electronic health records and through telephone verification with the caregiver to identify hospitalizations that were not captured by VA health records. A 2.5-day decrease in the number of days not at home during a 12-month period was hypothesized to be a clinically meaningful difference [25]. The second outcome was caregiver depressive symptoms measured by the Center for Epidemiologic Studies Depression Scale (CESD-10) at 12 months post randomization [26]. A research assistant administered the CESD-10 at baseline during the in-person enrollment meeting at the Durham VA and at 12 months over the telephone. Patients were censored if they entered a residential nursing home or residential psychiatric inpatient unit (defined as a stay of > 60 days) or at death; for details about sample size calculation, recruitment and attrition, unintended harms, and other aspects of study conduct see [18].

### Predictor measures

We assessed nine predictors that, based on existing evidence, were theorized to have an important moderating effect between the HI-FIVES intervention and our outcomes of interest [27–33]. Predictors included caregiver age, caregiver ethnicity (Hispanic vs. not), caregiver race (White vs. not), caregiver burden, caregiver depression, perceived financial difficulty (yes vs. no), relationship satisfaction, patient age, and patient medical complexity. These baseline measures were collected by the research assistant from the caregivers at an in-person enrollment meeting. Subjective caregiver burden was measured using the continuous Zarit Burden Scale in which higher scores indicated higher subjective burden [34]. Relationship satisfaction was measured using the continuous caregiver relationship subscale of the Caregiver Appraisal Scale in which higher scores indicate more satisfaction (range 1–55) [35, 36]. Nosos risk scores, a continuous index of patient complexity (higher score indicates more complexity [37]), takes into account the patients’ diagnoses (*ICD-9* codes), age, gender, and pharmacy records as well as VA-specific items such as VA priority status and VA-computed costs. In the model for days not at home, we also included caregiver baseline depressive symptoms measured continuously using the CESD-10 [26]. This self-reported measure of depression is calculated by summing the scores of 10 items (the range is from 0 to 30 with scores of 10 or more indicating depressive symptoms).

The trial registration number is: NCT01777490.

### Statistical analysis

We applied recursive partitioning methods to generalized linear models to construct decision trees by splitting nodes on the tree into daughter nodes to identify subgroups with substantially different effects from one another (https://cran.r-project.org/web/packages/partykit/vignettes/mob.pdf). Model-based recursive partitioning attempts to partition observations with respect to specific covariates and fit a local model in each cell of the partition. Score-based fluctuation tests the instability of the model’s parameters to determine the splits. Splitting ceases once the treatment-effect estimate is homogenous within each cell; in other words, the algorithm estimates no further differences in treatment effects based on the remaining parameters that have not yet been partitioned.

Analytical steps for model-based recursive partitioning are: (1) fit a parametric model to a dataset, (2) test for parameter instability over a set of partitioning variables, (3) if there is some overall parameter instability, split the model with respect to the variable associated with the highest instability, and (4) repeat the procedure in each of the daughter nodes [38].

Poisson (log link) and Gaussian distributions were used to model days not at home and caregiver depressive symptoms, respectively, at 12 months post randomization. Mean-centered stratification variables, patient cognitive and super-user status, were included. For the days-not-at-home model we included an offset for days observed (i.e., prior to censoring) and the stratification variables. The *glmtree* algorithm with default parameters in the *partykit* package in the R Statistical Environment was used. This algorithm preserves the randomized sample by examining combinations of interactions within treatment arm, which allowed us to estimate treatment effects under the assumption that observed and unobserved characteristics were similar across treatment and control arms. The models produced a glmtree for each outcome which we plotted and examined covariate balance across treatment arms within the identified subgroups using standardized mean differences (SMD); we used the convention of SMDs ≤ 0.2 to indicate an acceptable level of balance in small samples [39, 40].

We assessed the consistency of our results through 10-fold cross-validation on our sample and by comparing our results with other machine-learning algorithms that identify interactive effects. For the 10-fold cross-validation, the data was cut into 10 equally sized samples or folds; for each fold, the model was trained on 90% of the data and we assessed model fit—or how close predictions are to the observed values—in the remaining 10% of the data. A single glmtree is produced for each fold and so in addition to assessing model fit, we also examined the trees descriptively for variations in splits across folds compared with the tree built from the full dataset.

We applied two additional methods to verify whether other machine-learning algorithms might identify similar subgroups: mCART and random forest with interactions. The mCART approach was developed to improve balance among identified subgroups using RCT data; even when characteristics are balanced on the full sample, imbalance in subgroups may drive false detection of subgroup-specific effects [41]. mCART pair-matches treatment and control participants and estimates the treatment effect within each pair; a single tree is built to identify subgroups with differing treatment effects [41]. We also constructed a random forest (r*andomForestSRC*) that included all predictors and interactions between the treatment and each predictor [42]. We then examined the 95% confidence intervals for the variable importance of the interaction terms [43, 44]. The mCART algorithm does not accommodate count models, so we modeled the days-not-at-home outcome as a proportion of days not at home out of days in the study (count of days not at home/offset). The random forest models do not rely on linearity assumptions and so our outcomes were specified the same way as they were in the glmtree algorithm. We also examined a binary indicator of *any* days not at home using a classification tree and the  pair-matched algorithm.

Most variables had complete data; however, CESD-10 at 12 months was missing for *n* = 36 caregivers, nosos score was missing for *n* = 8 patients, and Zarit Burden score was missing for *n* = 2 caregivers. Most of the algorithms we used require complete data; therefore, we imputed the data for the variables above using adaptive tree imputations (*randomSurvivalForest* package) [43].

## Results

### Descriptive statistics

The total number of caregivers in the trial was 241; 118 in the intervention group and 123 in the control group. Patients on average were 73 (standard deviation (SD) = 11.7) years old. The sample was primarily non-Hispanic but was comprised of over 50% non-Whites, primarily African-Americans. Patients in the sample prior to censoring had a mean of 8.8 (SD = 13.8) days not at home over the 12 months post randomization; days not at home ranged from 0 to 80. Baseline caregiver Zarit Burden scores averaged 18.8 (SD = 9.7); caregiver baseline CESD-10 averaged 8.9 (SD = 5.9). Patients in this sample demonstrated substantial medical complexity; the mean nosos index was 3.4 (SD = 3.5). For additional details see Table 1.

**Table 1:** Descriptive characteristics

	HI-FIVES intervention arm (*n* = 118)	Usual care control arm (*n* = 123)
12-month days not at home, mean (SD)	8.91 (13.10)	8.62 (14.55)
12-month CESD-10 score, mean (SD)	8.17 (6.58)	7.58 (5.57)
Baseline CESD-10 score^a^, mean (SD)	9.15 (6.52)	8.78 (5.36)
Veteran age, mean (SD)	73.69 (11.24)	72.92 (12.12)
Caregiver age, mean (SD)	59.87 (11.78)	61.80 (12.60)
Caregiver White race (only^b^), %	56 (47.46)	48 (39.02)
Caregiver Hispanic, %	2 (1.69)	4 (3.25)
Relationship satisfaction score, mean (SD)	45.87 (5.79)	45.81 (5.71)
Zarit Burden score, mean (SD)	19.61 (10.04)	18.28 (9.30)
Perceived financial difficulty, %	61 (51.69)	49 (39.84)
Nosos comorbidity index, mean (SD)	3.44 (3.47)	3.71 (3.33)

^a^Statistics presented from imputed dataset; missing variables included CESD-10 at 12 months (*n* = 36), baseline nosos score (*n* = 8), and baseline Zarit Burden Scale score (*n* = 2)

^b^Does not include participants who indicated not White race or White plus another race category due to small cell size

### Days-not-at-home outcome

**Fig. 1:**
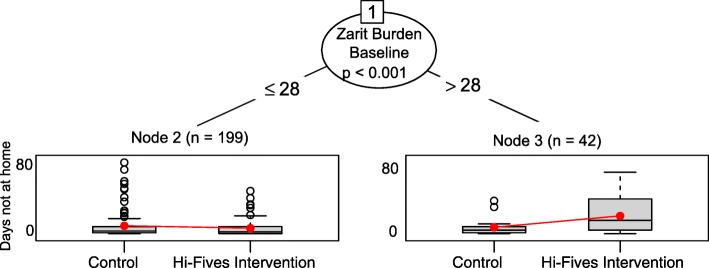
Glmtree algorithm for days not at home outcome

The glmtree algorithm identified statistically significant differences in treatment effects between caregivers with higher vs. lower Zarit Burden scores (cut-point identified by algorithm ≤ 28 vs. > 28; *p* = 0.01) (Fig. 1).

Specifically, patients of caregivers with a Zarit Burden score equal to or lower than 28 (*n* = 199) who participated in the HI-FIVES intervention had a 40% increase in days at home compared with patients whose caregivers did not participate in HI-FIVES. For patients of caregivers with a Zarit Burden score greater than 28 (*n* = 42), participation in HI-FIVES was related to a 63% decrease in the number of days at home. Note that decision trees only assess the statistical significance of differences in treatment effects between subgroups and do not provide confidence intervals for the effect estimates within subgroups. As a sensitivity check, we ran a Poisson regression model, with a similar specification to the model used for the glmtree algorithm, within each subgroup. In both subgroups treatment effects were statistically significant. However, given that this is an exploratory study and that our interest is in identifying subgroups, we do not focus on inferences about whether effect estimates represent a statistically significant difference between the treatment and control arms.

We identified several covariates that were not well-balanced across treatment groups in the subgroups (Table 2). Among the high Zarit Burden score group, White race (vs. black), and relationship satisfaction had SMDs higher than 0.20. Among the low Zarit Burden score group, financial difficulty was imbalanced. Nine out of 10 trees produced by folds of the data identified a single split on the Zarit Burden and showed similar trends in treatment effects among subgroups. One tree identified no subgroups. For the sensitivity analyses, the generalized linear trees found no subgroups when we looked at the effect on any days in the community. mCART identified no interactions between study arm and any covariates using the proportion of days not at home out of all days in the study. The random forest with interactions algorithm identified statistically significant variable importance values for several interaction effects, including, in order of importance, the Zarit Burden score (highest variable importance), the baseline CESD-10 score, the nosos score, and patient age (see Table 3).

**Table 2:** Covariate balance across subgroups identified by glmtree algorithm

	Outcome 1: days not in the community at follow-up						Outcome 2: caregiver depression scores at follow-up					
	Baseline Zarit Burden > 28			Baseline Zarit Burden ≤ 28			Baseline CESD-10 > 8			Baseline CESD-10 ≤ 8		
	Hi-Fives *n* = 23	Control *n* = 19	SMD	Hi-Fives *n* = 95	Control *n* = 104	SMD	Hi-Fives *n* = 54	Control *n* = 60	SMD	Hi-Fives *n* = 64	Control *n* = 63	SMD
Patient characteristics												
Baseline CESD-10 score, mean (SD)	15.04 (6.70)	15.47 (5.42)	0.07	7.73 (5.65)	7.56 (4.37)	0.03	–	–	–	–	–	–
Patient age, mean (SD)	71.74 (13.14)	72.05 (9.96)	0.03	74.16 (10.76)	73.08 (12.51)	0.09	72.07 (11.87)	72.18 (11.94)	0.01	75.05 (10.59)	73.62 (12.35)	0.12
Caregiver age, mean (SD)	59.65 (9.36)	59.05 (13.93)	0.05	59.93 (12.33)	62.30 (12.35)	0.19	58.72 (10.77)	62.05 (12.78)	**0.28**	60.84 (12.57)	61.56 (12.53)	0.06
White race, n (%)	14 (60.9)	7 (36.8)	**0.50**	42 (44.2)	41 (39.4)	0.10	28 (51.9)	24 (40.0)	**0.24**	28 (43.8)	24 (38.1)	0.12
Relationship satisfaction score, mean (SD)	41.83 (7.04)	40.42 (5.54)	**0.22**	46.85 (5.01)	46.78 (5.14)	0.01	43.48 (5.88)	44.17 (5.81)	0.12	47.89 (4.91)	47.35 (5.11)	0.11
Zarit Burden score, mean (SD)	–	–	–	–	–	–	26.15 (9.00)	21.82 (9.38)	**0.47**	14.09 (7.19)	14.88 (7.73)	0.11
Financial difficulty, *n* (%)	15 (65.2)	11 (57.9)	0.15	46 (48.4)	38 (36.5)	**0.24**	34 (63.0)	31 (51.7)	**0.23**	27 (42.2)	18 (28.6)	**0.29**
Nosos comorbidity index, mean (SD)	3.84 (4.42)	3.12 (2.57)	0.20	3.35 (3.18)	3.81 (3.35)	0.14	3.63 (3.93)	4.14 (3.74)	0.13	3.28 (2.98)	3.28 (2.65)	0.00

*SMD* Standardized mean differences; bolded SMD are greater than 0.2 and indicate poor balance; Statistics from imputed dataset; Super-user status, patient cognitive status, and caregiver Hispanic ethnicity were removed from the table because all cell sizes were smaller than 10

**Table 3:** Identified interactions across main models and sensitivity analyses

Outcomes	Models	Interaction effects	Type of analysis
Days not at home (count specification)	Generalized Poisson regression tree	Baseline Zarit Burden score (≤ 28, > 28)	Primary
	Random forest with interactions	Baseline Zarit Burden score, CESD-10, nosos score and patient age	Sensitivity
Proportion of days not at home	mCART regression tree	None	Sensitivity
Any days not at home	Generalized linear regression tree (binomial)	None	Sensitivity
	mCART classification tree	None	Sensitivity
CESD-10 score 12-month follow-up	Linear regression tree	Baseline CESD-10 score (≤ 8, > 8)	Primary
	mCART regression	None	Sensitivity
	Random forest with interactions	Baseline CESD-10 score and, Zarit Burden score	Sensitivity

### Caregiver depressive symptoms outcome

**Fig. 2:**
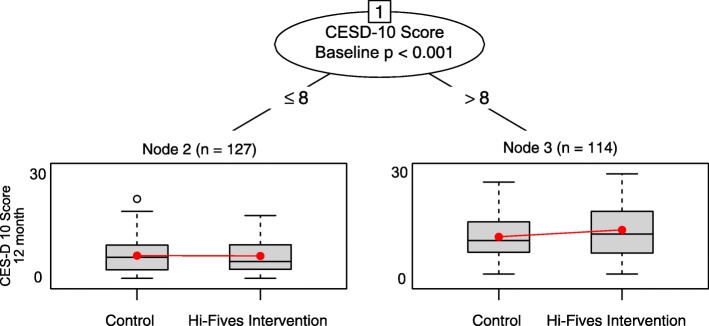
Glmtree algorithm for caregiver depressive symptoms outcome

The recursive partitioning algorithm identified one split on depressive symptoms and produced two daughter nodes (cut-point identified by algorithm ≤ 8 vs. > 8 on baseline CESD-10; *p* = 0.01) (Fig. 2). Caregivers with a baseline CESD-10 score of 8 or lower (*n* = 127) who participated in HI-FIVES experienced an average decrease in 0.06 points on the CESD-10 score at 12 months post intervention. Caregivers with a score greater than 8 (*n* = 114), on average, had a 1.5 higher CESD-10 score at the end of follow-up. As a sensitivity check, we also ran the linear regression models within each subgroup, neither treatment-effect estimate was statistically significant. However, our interest is in identifying subgroups and not assessing within-subgroup treatment effects.

We identified several covariates that were not well-balanced across treatment groups in the subgroups (Table 2); among the higher baseline CESD-10 score, caregiver age, Zarit Burden score, and financial difficulty had SMDs greater than 0.20. Participants in the lower baseline CESD-D score subgroup were not well-balanced on patient age, Hispanic ethnicity, and perceived financial difficulty.

Across folds of the data, all 10 glmtree algorithms identified a single split on baseline CESD-10 score and trends in treatment effects among subgroups were similar. For the sensitivity analyses, mCART identified no interactions. The random forest algorithm identified interactions between study arm and baseline CESD score and Zarit Burden score (see Table 3).

## Discussion

This study demonstrates how to use machine-learning algorithms with data from RCTs to explore potential subgroup effects that may be masked when trials examine outcomes as average treatment effects. We compare several algorithms, including a glmtree algorithm (primary analysis), mCART (sensitivity analysis), and random forest with interactions (sensitivity analysis). This is the first post-hoc analysis that uses machine learning to examine heterogeneous treatment effects of an intervention for informal caregivers.

The algorithm identified a cut-point of 28 on the Zarit Burden Scale—clinically significant burden is 18 and above [45]—therefore, a score of 28 and above (*n* = 42) represents a group of extremely distressed caregivers. For the CESD-10 outcome, the algorithm identified a cut-point of 8 (CESD-10 > 8 *n* = 114), which aligns with clinical standards for probable depression [26]. For both outcomes, we only identified one subgroup with differential treatment effects which suggests that these characteristics uniquely drove risk. Our sensitivity analyses also provide support for baseline caregiver burden and depressive symptoms as potential moderators of the relationship between treatment and both of our outcomes at 12 months post intervention.

We did not test the statistical significance of the within-subgroup treatment-effect estimates in the model-based glmtree algorithms because we did not have a large enough sample to train our model and then validate the findings in a test dataset. However, the treatment effects identified by the glmtree algorithm suggest that caregivers with higher baseline levels of burden and depression may not have been helped by the caregiver skills and education intervention. These findings must be replicated, but it is possible that low-intensity, short-term interventions are not enough to help highly distressed and burdened caregivers. In fact, individualized or one-on-one interventions that target a specific outcome may be required make substantial improvements [16].

### Limitations and considerations

Our study also highlights the challenges of applying machine learning to health services research, in general, and to post-hoc analyses of clinical trials, in particular [23, 46]. There are notable limitations both in the methods and in the programs available to implement the methods. First, machine-learning methods do not require large sample sizes and are known to work well for datasets with many predictors relative to observations. However, small samples (*n* < 400) [47] may pose limitations because there are not enough observations to train and test algorithms and to produce fit statistics for the main model. This is a major challenge for post-hoc analyses of trial data because most intervention trials in health care have relatively small samples. The work that has been done to date to apply machine learning to trial data has taken advantage of large health trials [23, 46]. To address this challenge with our small sample we examined the consistency across folds of our dataset; our trees were consistent.

Another challenge related to small sample size is that we were unable to generate measures of variability for subgroup level treatment-effect estimates. The glmtree algorithm that we used provided a measure of statistical significance indicating whether or not there were differences in treatment-effect sizes between subgroups and not whether the treatment effect itself was statistically significant within subgroups. While the model output provided a within-subgroup treatment-effect estimate, it did not provide a measure of variability of the effect estimate. Therefore, we only report these estimates and not associated confidence intervals because we did not have a large enough sample to train the glmtree algorithm and run this algorithm on a validation dataset to generate standard errors for the estimates. However, the goal of our analysis is to explore potential heterogeneous treatment effects and not to report treatment effects by subgroup.

Second, an inherent problem with single decision trees is that they tend to overfit the data [48]. In addition, simulation studies suggest that characteristics within identified subgroups may not be balanced across treatment and control groups, even if characteristics are balance on the full sample, which could falsely induce subgroup identification [41]. Indeed our subgroups were not fully balanced on baseline covariates (Table 2). To attempt to address the potential limitations of overfitting and poor balance, we examined the trees across folds of the data (described above) and ran several analyses to test the robustness of the results. First, we used the mCART approach—which balances on matched pairs and thus ensures that subgroups identified by the decision tree are balanced. We also searched for interactive effectives using a random forest with interactions algorithm. While the mCART algorithm did not identify subgroups for the days-not-at-home outcome, the random forest model did identify an interaction between treatment and baseline CESD-10. mCART is inefficient for small samples [41], which may explain why we did not identify any subgroups using this method for the days-not-at-home outcome. Because of this, we also applied a virtual twin approach [49], which is not bounded by linearity assumptions; the results using this approach confirmed our results from the main analyses for both outcomes.

Statistical environments, including R and Python, offer the most variety of machine-learning packages, yet package development in these environments is user-driven. As machine learning is just starting to be used for health services research, many of the existing packages do not accommodate outcome specifications commonly used in the field. mCART does not accommodate count outcomes and, therefore, we modeled prevalence of days not at home using a linear model with normal distribution which would have been more likely to produce biased variance estimates; modeling the data using Poisson regression could have led to more efficient and accurate estimates. Different outcomes specifications (i.e., count of days vs. proportion of days) may be another reason why our sensitivity analyses did not identify subgroups.

### Research implications

We attempted several approaches to limit the impact of these external limitations. Our goal was to identify subgroups with heterogeneous treatment effects to help future caregiver interventionists better target their population. While we were unable to fully overcome these limitations, we offer a novel approach and considerations for other researchers who wish to conduct post-hoc trial analyses. For researchers who are designing interventions for highly burdened and distressed caregivers, a tailored, more intensive intervention that involves assessing caregiver needs and developing personalized approaches may be warranted. However, caregivers with lower levels of burden and depression may benefit from a group and telephone-based skills training program, such as HI-FIVES.

## Conclusions

Using model-based recursive partitioning methods to conduct a post-hoc analysis of subgroup effects of the HI-FIVES intervention, we found potential evidence for heterogeneous treatment effects. In general, use of these methods can be constrained by limitations that are common in RCTs of clinical interventions, including small sample sizes and outcomes that do not meet the distributional assumptions of machine-learning algorithms in existing software programs. We present a process for applying these methods using data with such limitations and suggest various sensitivity analyses and robustness checks. Further, we demonstrate how our results can be used for hypothesis generation as opposed to inference about subgroup effects.

## Data Availability

The datasets generated and/or analyzed during the current study are not publicly available because individual privacy may be comprised and we do not have permission to share this private data, but analytical models and code are available from the corresponding author on reasonable request.

## Abbreviations

CESD-10: Center for Epidemiologic Studies Depression Scale
ED: Emergency department
HCBS: Home and community-based services
HI-FIVES: Helping Invested Family Members Improve Veteran’s Experiences Study
SD: Standard deviation
SMD: Standardized mean differences
SPMSQ: Mental Status Questionnaire
VHA: Veteran Health Administration
